# Inflammation and the Gut-Liver Axis in the Pathophysiology of Cholangiopathies

**DOI:** 10.3390/ijms19103003

**Published:** 2018-10-01

**Authors:** Debora Maria Giordano, Claudio Pinto, Luca Maroni, Antonio Benedetti, Marco Marzioni

**Affiliations:** Clinic of Gastroenterology and Hepatology, Università Politecnica delle Marche, Via Tronto 10, 60126 Ancona, Italy; deboramariagiordano@gmail.com (D.M.G.); pintoclaudio86@gmail.com (C.P.); luca.maroni@live.it (L.M.); Antonio.Benedetti@osepdaliriuniti.marche.it (A.B.)

**Keywords:** cholangiocytes adaptive and immune response, cholangiopathies, leaky-gut hypothesis, PSC and PBC-microbiota signature

## Abstract

Cholangiocytes, the epithelial cells lining the bile ducts, represent the unique target of a group of progressive diseases known as cholangiopathies whose pathogenesis remain largely unknown. In normal conditions, cholangiocytes are quiescent and participate to the final bile volume and composition. Following exogenous or endogenous stimuli, cholangiocytes undergo extensive modifications of their phenotype. Reactive cholangiocytes actively proliferate and release a set of proinflammatory molecules, which act in autocrine/paracrine manner mediating the cross-talk with other liver cell types and innate and adaptive immune cells. Cholangiocytes themselves activate innate immune responses against gut-derived microorganisms or bacterial products that reach the liver via enterohepatic circulation. Gut microbiota has been implicated in the development and progression of the two most common cholangiopathies, i.e., primary sclerosing cholangitis (PSC) and primary biliary cholangitis (PBC), which have distinctive microbiota composition compared to healthy individuals. The impairment of intestinal barrier functions or gut dysbiosis expose cholangiocytes to an increasing amount of microorganisms and may exacerbate inflammatory responses thus leading to fibrotic remodeling of the organ. The present review focuses on the complex interactions between the activation of innate immune responses in reactive cholangiocytes, dysbiosis, and gut permeability to bacterial products in the pathogenesis of PSC and PBC.

## 1. Introduction

The biliary tree is composed of a tridimensional network of ducts that drain the bile into the lumen of the intestine and are lined by epithelial cells termed cholangiocytes [1,2]. Cholangiocytes are a heterogeneous cell population from both a functional and a morphological point of view. Cholangiocytes are divided into small and large cholangiocytes, which encircle small and large intrahepatic bile ducts, respectively. Large cholangiocytes actively participate to modification of bile composition and volume through secretory and absorptive mechanisms tightly regulated by the action of different molecules (e.g., hormones, peptides, and neurotransmitters) [3]. On the other hand, small cholangiocytes are able to modify their phenotype in response to exogenous/endogenous noxious stimuli (e.g., microorganisms, toxins/drugs, or hormones), thus participating to the inflammatory response during biliary tree damage [3,4], and serve as liver progenitor cells under certain conditions [5]. Cholangiocytes are the central target of a group of diseases known as cholangiopathies with different etiologies (e.g., genetic, autoimmune, infectious, drug-induced, idiopathic malignant, secondary sclerosing cholangitis). In response to injury, cholangiocytes that are in a quiescent state enhance proliferation and start to release mediators through which they interact with both resident and non-resident liver cells, maintaining tissue homeostasis and function, and contributing to immune cells and myofibroblasts chemotaxis at the damage site.

In health conditions, an enormous amount of gut-derived molecules reaches the liver via the portal blood circulation without eliciting any inflammatory response. In disease state, when the intestinal barrier function is defective or in case of modifications of gut bacterial homeostasis, an altered composition of gut-derived products reaches the liver where it may induce or exacerbate hepatic inflammation eliciting complex responses in hepatic cells including cholangiocytes. 

An increasing body of clinical and experimental data has highlighted the prominent role of an altered-gut microbiota in the onset and perpetuation of a subgroup of chronic cholestatic liver diseases, particularly of primary sclerosing cholangitis (PSC) and primary biliary cholangitis (PBC). Both diseases are characterized by portal inflammation, bile duct damage, and subsequent fibrosis development, and progress to cirrhosis leading to end-stage liver failure. Despite these disorders share common features, PSC targets mainly medium to large extrahepatic and intrahepatic bile ducts while PBC is an autoimmune disease characterized by serum positivity to antimitochondrial antibody (AMA) and damage of small intrahepatic bile ducts [6,7]. Thus, awareness of biliary epithelium heterogeneity is function to understand the molecular mechanisms underlying the pathophysiology of cholangiopathies and possibly to devise new selective therapeutic targets. Ursodeoxycholic acid (UDCA) administration is currently the primary therapeutic approach for the treatment of PBC patients [3]. The 40% of patients who do not respond to UDCA, are subjected to obeticholic acid (OCA) administration. Therefore, the combination of the two existing therapies is not effective in whole PBC patient’s treatment. By contrast, up until now, no approved therapies for PSC patients exist. The only therapeutic strategy is liver transplantation. Several clinical trials based on the administration of drugs targeting specific cholangiocytes pathways, such as agonists of nuclear receptors and membrane transporters involved in bile acids homeostasis and metabolisms, are currently ongoing [8].

## 2. Cholangiocyte Adaptation to Injury

In response to exogenous/endogenous damage, cholangiocytes undergo noteworthy modifications of their phenotype and become “reactive”. In this setting, biliary epithelial cells release a wide range of proinflammatory and fibrogenic mediators orchestrating cholangiocyte proliferation, senescence, and apoptosis as well as immune and mesenchymal cells chemotaxis to repair the injured tissue and remodel the biliary tree [3,9]. These events determine cholangiocyte proliferation, which is intended to compensate for the anatomic loss of biliary cells and also to sustain secretory activities, tissue infiltration of immune cells, deposition of matrix proteins and activation of liver progenitor cells [10]. Unless reversed, these mechanisms lead to periportal fibrosis and ductopenia, when the balance between cell proliferation and cell death events is lost and, eventually, to biliary cirrhosis [11,12]. Cholangiocytes differentially proliferate in response to liver injury and toxins. Several stimuli, including bile acids, acetylcholine, estrogen, hepatocyte growth factor, and IL-6 may induce cholangiocyte proliferation by binding to specific receptors. Antiproliferative mediators such as somatostatin, gastrin, and interferon γ (INFγ) are also known [13,14]. 

Along with proliferation, cholangiocyte response to injury is characterized by the so-called “neuroendocrine-like transdifferentiation”, which plays an essential role in immune responses, hepatic inflammation and development of liver fibrosis in addition to sustaining biliary proliferation itself [4,15]. Indeed, activated cholangiocytes express and secrete various proinflammatory cytokines and chemokines (e.g., IL-6, IL-8, TNFα, and various growth factors) and respond to signaling cascades such as Notch and Hedgehog [16]. 

The signaling pathway of the gastrointestinal peptide hormone secretin (SCT), which binds the secretin receptor (SR), is one of the most studied in cholestatic liver injury. It has been supposed that the SCT/SR axis is associated with cholestatic liver injury because rats show elevated expression of SR after bile duct ligation (BDL) [17]. When the SCT binds to SR, which is expressed only in the basolateral membrane of large cholangiocytes, its activation elevates intracellular adenosine 3,5′-cyclic monophosphate (cAMP) levels leading to enhanced cell proliferation, exocytosis, and ductular secretion in cholangiocytes [18,19]. The increase in intracellular cAMP levels induces bicarbonate secretion from cholangiocytes into the bile [20], and the activation of ERK1/2 and Elk-1 signaling pathway [21,22]. Since small cholangiocytes do not express CFTR and SR, only large cholangiocytes are responsible for CFTR- or SR-dependent proliferation and bicarbonate secretion in the liver, and are involved in SCT-induced ductular secretion [23]. 

In small cholangiocytes, Ca^2+^ signaling is more important for proliferation. During histamine-induced small cholangiocyte proliferation, inositol 1,4,5-trisphosphate (IP3) levels are increased [24]. The binding of IP3 to IP3 receptor (IP3R), activates the release of Ca^2+^ into the cytosol and the subsequent activation of calcineurin (CN) and calmodulin (CaM). An elegant study showed that CN mediates the phosphorylation of the nuclear factor of activated T-cells (NFAT) proteins [25], while activation of CaM leads to the activation of CaM-dependent kinase (CaMK) [26]. In this way, small cholangiocytes proliferate through IP3/Ca^2+^/CaMK signaling [24,27]. Furthermore, the activation and nuclear translocation of NFAT2 increases in proliferating small cholangiocytes [28]. This pathway has been also identified in hepatocellular carcinoma contributing to the maintenance of cell proliferation [29]. In large cholangiocytes, Ca^2+^ signaling may not be critical for cell proliferation during cholestatic liver injury, since expression levels of IP3R are decreased in large cholangiocytes during BDL [30].

Although the cAMP pathway may be more important than the IP3/Ca^2+^ pathway for large cholangiocyte proliferation, Ca^2+^ signaling is essential for ductular secretion in large cholangiocytes [23], and a study has demonstrated that trigger-induced Cl^−^ secretion is Ca^2+^ dependent [31]. An in vivo experiment carried out on rats showed that the expression of SR and CFTR as well as intracellular cAMP levels and cholangiocyte proliferation, were increased after SCT administration [32]. Moreover, SCT is expressed by cholangiocytes and S cells which are located primarily in the mucosa of the duodenum [33]. High levels of SCT expression were found in both large cholangiocytes and S cells during BDL [34]. In contrast, SCT knockout (SCT^−/−^) mice and SR^−/−^ mice show an attenuated bile duct hyperplasia after BDL compared to wild-type mice [34,35]. 

Another recent study has highlighted a new possible mediator in cholangiocyte response to damage. In particular, it has been shown that cholangiocytes express gonadotropin-releasing hormone (GnRH) receptor 1 (GnRHR1) and its expression levels are elevated during BDL in rats [36]. GnRH is a tropic peptide hormone that modulates cell proliferation and act, depending on the cell type, by stimulating or inhibiting the proliferation [37]. Administration of GnRH increases bile duct mass and intracellular cAMP levels, expression of SR, CFTR, and AE2 in cholangiocytes in vivo. On the other hand, in vivo knockdown of GnRH decreased intrahepatic bile duct mass and fibrosis induced by BDL [36]. Moreover, enhanced GnRH serum levels as well as GnRHR1 expression in the liver, were observed in human PSC patients compared to healthy individuals [38]. 

A different known mediator of the cholangiocyte response to damage is the neuropeptide substance P (SP). The binding of SP to neurokinin-1 receptor (NK-1R) increases cytokine expression thus leading to the inflammatory response [39]. Patients with chronic liver disease show increased serum levels of SP compared to respective controls, also reproduced in cholestatic rat models [40]. Elevated SP secretion is also identified in cholangiocarcinoma (CCA) cell lines and treatment with an antagonist inhibits proliferation of CCA cells [41]. Moreover, the increase of SP serum levels and NK-1R expression in the liver were observed in human PSC patients compared to healthy group [42]. Elevated NK-1R expression was found in large cholangiocytes of BDL mice, and NK-1R^−/−^ mice show attenuated large cholangiocyte proliferation and bile duct hyperplasia [43].

More and more new studies appear to deepen the role of some possible mediators of the cholangiocyte adaptation to injury. The role of histamine is already known in the induction of small and large cholangiocyte proliferation, where agonists for H1 histamine receptor induce small cholangiocyte proliferation, and agonists for H2 induce large cholangiocyte proliferation [24,27]. The inhibition of mast cell-derived histamine secretion has been shown to attenuate bile duct hyperplasia during BDL in vivo [44]. On the other hand, during cholestatic liver injury such as PBC, the number of mast cells in the liver is increased, probably due to their role in histamine secretion [45]. 

Another possible mediator is the melatonin, a hormone produced by the pineal gland, small intestine and liver [46]. An in vivo experiment carried out on BDL rats showed that the melatonin administration reduces liver fibrosis and serum cytokine levels for IL-1β and IL-6 [47], but it has recently been shown that melatonin administration inhibits GnRH secretion and reduces bile duct hyperplasia and liver fibrosis in BDL rats [48]. Other studies indicate that numerous other pathways, such as pancreatic duodenal homeobox-1 (PDX-1), vascular endothelial growth factor (VEGF) A/C and relative receptor VEGFR-2/3, and galanin and galanin receptor 1 (GalR1) are associated with cholangiocyte proliferation and liver fibrosis in cholestatic liver disease such as during BDL in rats [49,50].

A relevant role in shaping cholangiocyte response to injury is played by genetic variants, epigenetic mechanisms, and post-transcriptional phenomena (including the influence of microRNAs on protein expression [51]), which together determine whether reactive cholangiocytes regress to a normal phenotype or lead to chronic inflammation of the bile duct, with progression of the cholangiopathy. An elegant study of Lazaridis and LaRusso [11], summarized the recent findings concerning the individual genetic variants which may be implicated in cholangiopathies onset and disease progression. Data collected by four different genome wide-association studies (GWASs) have unveiled the presence of 30 susceptibility loci associated with PBC related to immune cells functions and processes. With regards to PSC, 31 risk loci have been identified by GWASs [52]. However, nine of these susceptibility loci do not reach the genome-wide significance [52]. As for PBC, PSC-associated risk loci are involved in innate and adaptive immune cells responses. In addition, genetic variants of fucosyltransferase 2 (FUT2), described in detail beyond, have been associated to gut microbiota modification in PSC patients [53].

Further investigations are needed to delineate the role of genetic factors in the pathophysiology of cholangiopathies; these studies prove to be complex also because of the difficulty in establishing the environmental contribution to the progression of the disease. With this regard, the recognition of the heterogeneity of cholangiocytes along the biliary tree is of fundamental importance, in order to better understand the cholangiopathies [54,55].

## 3. Innate Immune Response Activation

Biliary epithelial cells represent the first line of defense of the biliary system against microbial products and other potential noxious mediators that are continuously translocating from the gut to hepatic sinusoids [16]. Cholangiocytes are provided with pathogen recognition receptors (PRRs), adaptor proteins and related signaling pathways. PRRs recognize and bind to bacterial components known as pathogen-associated molecular patterns (PAMPs) such as lipopolysaccharide (LPS) or bacterial DNA fragments. Molecules released in the extracellular environment from damaged cells defined as damage-associated molecular patterns (DAMPs) can also activate PRRs of cholangiocytes [56]. The best characterized PRRs family is the Toll-like receptors (TLRs) consisting of a number of type 1 transmembrane glycoprotein receptors (10 members in human and 13 in mammalian cells) activated in response to specific conserved components shared among microorganisms [57]. Exposure of cholangiocytes to LPS leads to TLR4 activation, which in turn triggers intracellular signals culminating in the activation of nuclear factor-kappa B (Nf-κB) or activator protein-1 (AP-1) [58,59], genes involved in the biosynthesis of proinflammatory cytokines [60]. Beside TLRs family, the nucleotide-binding oligomerization domain (NOD)-like receptor (NLR) family consists of soluble PRRs, which sense intracellular pathogens and endogenous dangerous stimuli through the DAMPs [61]. Based on N-terminal structures, NLR family proteins are classified into different subfamilies [62]. Among these, the nucleotide-binding oligomerization domain (NOD)-like receptor (NLR) family, pyrin domain-containing protein 3 (Nlrp3) has been recently investigated and described as a member of innate immune system activated by both PAMPs and DAMPs. Nlrp3 is assembled into a multiprotein complex known as the “inflammasome” which, upon activation, triggers the proteolytic cleavage of cytokine precursors mediated by caspase-1 [61,63], leading to the secretion of IL-1β and IL-18 in the mature and biologically active form [56]. 

**Figure 1:**
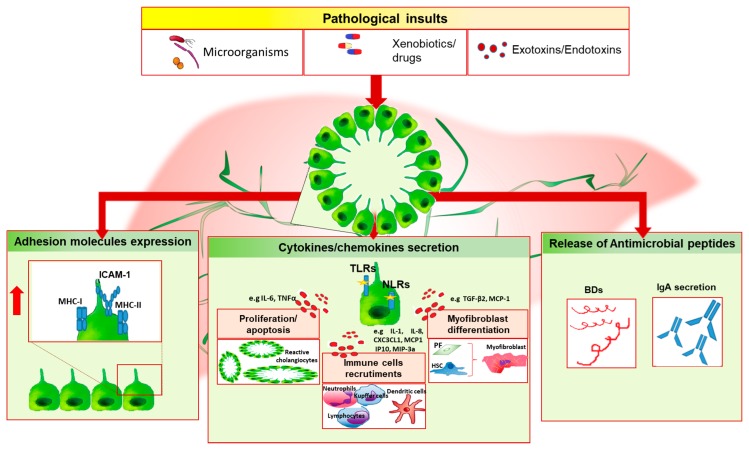
Cholangiocyte immune response. Biliary epithelial cells are exposed to different dangerous stimuli such as microorganisms, drugs or toxins, and others exotoxins/endotoxins which trigger tissue damage. In this setting, cholangiocytes may modify their biology and phenotype by increasing the release of proinflammatory mediators (e.g., IL-6, IL-8, IL-1), which act in autocrine/paracrine fashion on both resident and non-resident cells, by upregulating the expression of surface proteins (i.e., MHC-I, MHC-II and ICAM-1) and by releasing antimicrobial molecules such as beta-defensins (BDs) or IgA following epithelial transcytosis. These events lead to ductules’ proliferation, immune cells chemotaxis and myofibroblast differentiation. In case of persistent biliary damage, these processes could lead to chronic inflammation and fibrosis establishment. PF, portal fibroblast; HSC, hepatic stellate cell.

Our group of research has recently shown that the Nlrp3 inflammasome is upregulated in injured cholangiocytes, both in a murine model of sclerosing cholangitis and in biliary cells of PSC patients. In vitro, activation of Nlrp3 induced the expression of IL-18 and influenced the epithelial barrier function of cholangiocytes by blunting the increased epithelial permeability stimulated by LPS incubation [64]. Reactive cholangiocytes actively participate to host immune response by releasing a panel of cytokines and chemokines, which act in autocrine and paracrine fashion on resident liver cells and immune cells thus increasing the expression of surface adhesion molecules and producing antimicrobial peptides, which directly exert host defense activities (Figure 1).

Following an initial insult, cholangiocytes secrete IL-6, which determines cholangiocyte proliferation to compensate biliary mass loss and preserve biliary epithelial cells functions. LPS, TNFα, or IL-1 trigger the releasing of IL-8, the epithelial cell derived neutrophil activating protein (ENA-78) and growth-related oncogene (GRO), which mediate neutrophil recruitment at the inflammation site [65]. Differently, dendritic cells chemotaxis is driven by cholangiocyte secretion of macrophage inflammatory protein-3a (MIP-3a) following IL-1β, TNFα, IL-17 stimulus, or TLRs activation [66]. The chemoattraction ability of biliary epithelial cells comprises the releasing of the C-X-3-C motif chemokine ligand 1 (CX3CL1), also known as fractalkine. This chemokine act as chemoattractant molecule in its soluble form by promoting monocytes recruitment and lymphocytes T homing, and as adhesion molecule in its membrane-bound form, by mediating the adhesion of leukocyte-expressing the chemokine receptor (CXC3CR1) to endothelium [67]. IL-6 also exerts a pleiotropic effect by promoting the terminal differentiation of lymphocytes B cells, which secrete serum immunoglobulins [68]. A subtype of epithelial inflammatory cytokines modulate the expression of adhesion molecules on the surface of cholangiocytes, which serve for cell-mediated immune response. In vitro experiments have shown that TNFα, IL-1, and INFγ drive the upregulation of the adhesion molecules MHC-I, MHC-II, and ICAM-1 on cholangiocyte membrane [69,70]. Conversely, the negative regulation of this mechanism is mediated by TGFβ, which decreases the expression of these surface proteins [71]. Alternatively to MHC-I, CD8^+^ lymphocytes activation during biliary injury can be triggered by the upregulation of CD40 ligand on cholangiocytes surface upon INFγ or TNFα stimulus [71]. 

During chronic injury of the bile ducts, the array of proinflammtory mediators released undergoes extensive modifications. Following INFγ stimulation, cholangiocytes increase the secretion of monocyte chemoattractant protein-1 (MCP-1), monokine induced by INFγ (Mig), interferon inducible T cell alpha chemoattractant (ITAC) and interferon gamma inducible protein-10 (IP-10) [65]. Although MCP-1 is a well-known chemotactic factors for monocytes and lymphocytes, it also mediates fibrogenic effects by stimulating portal fibroblast differentiation and collagen-I secretion, and by inducing HSC homing [72,73]. With this regard, proliferating cholangiocytes upregulates MCP-1 expression whose levels correlate with a poor prognosis of disease [74]. One of the key features of chronic biliary injury is represented by fibrogenesis, which involves the participation of different cell subtypes. Biliary epithelial cells increase the releasing of a multifunctional cytokine, the TGFβ; in particular, high levels of β2 transcripts have been detected in reactive biliary cells of CCl_4_-treated fibrotic rat livers, while no expression has been evidenced in the other cell subtypes [75]. TGFβ induces the differentiation of portal fibroblast, the release of Endothelin-1 by cholangiocytes and the inhibition of biliary epithelial cells proliferation [76,77].

Bile duct loss is a consequence of chronic biliary inflammation. Co-incubation of TNFα and Actinomycin D determines cholangiocyte apoptosis and loss of secretion capabilities in BDL rat, suggesting that during cholestasis reactive cholangiocytes are more susceptible to TNFα cytotoxic effect [78]. Accordingly, the administration of taurocholate prevents TNFα-induced cholangiocyte damage through the PI3K molecular pathway activation [79]. In addition, in experimental biliary atresia it has been shown the ability of TNFα to promote cholangiocyte programmed cell-death through the activation of TNFα/TNFR2 molecular pathway [80].

In vitro studies have shown that murine and rat cholangiocytes treated with conditional medium collected by myofibroblast hepatic stellate cells, increase the production of soluble mediators involved in immune cell chemotaxis, antigen presentation and processing via activation of Hedgehog (Hh) signaling, as confirmed by Hh-neutralizing antibody challenge [81]. The upregulation of these immune mediators has been confirmed in vivo in patched (ptc) haplo-insufficient (*ptc*^+/−^) mice (with impaired capacity to deactivate Hh signal) subjected to BDL, a model of biliary fibrosis associated with liver Hh pathway activation [81,82]. Previous studies demonstrated the ability of biliary epithelial cells and hepatic stellate cells to express and respond to Hh ligands [83,84]. The perpetuation of Hedgehog pathway activation has been related to biliary mass loss and fibrosis due in part to the induction of epithelial-mesenchimal transition of hepatic progenitors [85] and in part to myofibroblastic phenotype retention through an autocrine mechanism [84,86]. 

Induction of biliary senescence has been recently related to cholangiocyte immune response deregulation during cholangiopathies. PSC cholangiocytes show an increased expression of cellular senescence markers and of the senescence-associated secretory phenotype (SASP) [87]. Cholangiocytes from multi-drug resistance 2 knockout (mdr2^−/−^) mice, a model of PSC, undergo senescence [87]. Mdr2 is a canalicular phospholipid flippase encoded by the abcb4 gene, involved in the transport of phospholipids into bile. At this level, phospholipids assemble together with bile acids in mixed micelles that prevent the damage of biliary epithelium induced by toxic bile acids [88]. In the mouse model, the absence of mdr2 causes bile duct injury mediated by free bile acids which results in sclerosing cholangitis development. Lesions are characterized by portal inflammation, onion-skin lesions and obliterative cholangitis resembling PSC hallmarks [88]. As mentioned above, cholangiocytes are equipped of TLRs family receptors through which recognize PAMPs. The activation of TLR4 by transient LPS stimulus rapidly determines N-Ras activation and subsequently induces downstream events culminating in cholangiocyte IL-6 secretion [89]. Conversely, human cultured cholangiocytes subjected to persistent LPS treatment acquire a senescent phenotype characterized by the upregulation of proteins involved in cell cycle arrest (p16I^NK4a^ and p21^WAF1/Cip^) and the expression of SASP components [87]. Despite the precisely mechanism of cholangiocyte senescence is poorly understood, these fascinating results suggest an important role of non-replicative cholangiocytes in the modulation of biliary microenvironment and organ homeostasis [87,90]. Senescent cholangiocytes contribute to progressive ductopenia due to the loss of proliferative and regenerative capabilities that make non-replicative cells prone to subsequent injuries with worsening inflammation [87]. 

A novel attracting mechanism through which cholangiocytes mediate intercellular communication in the setting of microbial infection has been recently reported. The extracellular vesicles (EVs)-mediated cell–cell communication has been demonstrated in cultured immortalized cholangiocyte H69 cell lines following LPS challenge [91]. Endotoxin treatment determines an increase of LPS-derived EVs secretion in immortalized human cultured cholangiocyte. Cultured cholangiocytes stimulated with LPS-derived EVs enhance the inflammatory response by upregulating mRNA levels of proinflammatory cytokines. Knock-down of either SCT and SR determines a reduction of EVs released by large cholangiocyte following LPS treatment. Moreover, EVs collected by STC and SR knocked-down large cholangiocytes do not elicit an inflammatory response in control large cholangiocytes. By taking into account these data, it is possible to hypothesize that inflammatory mediators secreted in response to LPS-derived EVs and influenced by the STC/SR axis could act on immune cells to contain biliary damage [91]. 

Cholangiocyte immune capability relies also on secretory immunoglobulin A (sIgA) transcytosis into the bile to preserve mucosal integrity during pathogens infection. The opsonization of circulating bacterial antigen mediated by sIgA prevents microbial attachment on cholangiocyte surface [92]. The secretory capacity of cholangiocyte includes the synthesis of antimicrobial peptides. Defensins are small proteins rich in cysteine that bind to the microbial surface inducing membrane disruption, intracellular ion release and ultimately cell death. Of the two existing subfamilies, α and β-defensin, the latter serves as mucosal defense against local infections. In humans six isoforms (Hbd-1 to Hbd-6) have been identified. In vitro, the Hbd-1 is constitutively expressed in the cytoplasm of cultured biliary epithelial cells lining the intrahepatic bile ducts; instead, the expression of Hbd-2 occurs upon LPS challenge, *Escherichia coli* infection [93] or stimulation by TNFα and IL-1β whose levels are reported to be increased in cholangiopathies in the portal tract [94].

## 4. Leaky-Gut Hypothesis

**Figure 2:**
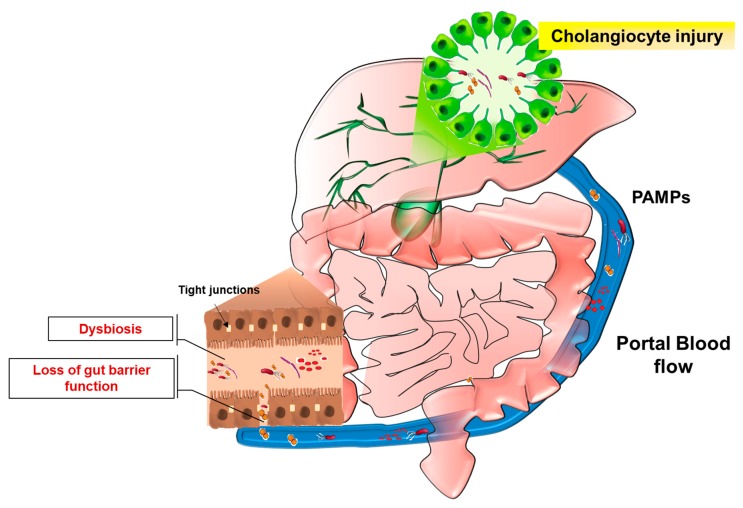
The leaky-gut hypothesis. Portal blood flow ensures the metabolic connection between intestine and liver. During gut inflammation, which leads to impairment of intestinal barrier functions (e.g., downregulation of tight junctions), or as a consequence of dysbiosis establishment, a considerable amount of microorganisms and related molecules (PAMPs) reach the liver and may determine hepatobiliary inflammation. In this context, deregulation of cholangiocyte response to injury is thought to contribute to disease onset and progression.

The term “gut microbiota” refers to commensal and pathogen bacteria, and other microorganisms (i.e., Fungii, viruses), including metabolites, bacterial products or genomes, that colonize the intestinal tract [95]. The gut-colonizing microorganisms are in symbiosis with the host and possess functional roles in human metabolism, (e.g., by production of vitamins or secondary biliary acids) [96]. The lack of this beneficial association is known as dysbiosis and it depends on qualitative changes of gut microbiota, due to the increased extent of pathogen bacteria with respect to commensal flora, or to quantitative changes caused by bacterial overgrowth [97]. In health conditions, the gastrointestinal tract exhibits physical (intestinal barrier and mucus layer) and chemical barriers (IgA or antimicrobial peptides and proteins) that prevent the translocation of bacteria towards the liver. A portion of gut microbial antigens enters in the portal blood circulation and is recognized by liver immune cells without eliciting an immunological response because of the existing mechanism of “immune tolerance” [98,99]. The impairment of intestinal barrier functions observed during gut inflammation exposes the liver to an increasing number of bacteria, bacterial components or metabolites, which may trigger hepatic inflammation and fibrotic remodeling (Figure 2).

These notions establish the “leaky-gut” hypothesis which may contribute to the pathogenesis of hepatobiliary diseases.

A clear association between intestinal inflammation, gut microbiota modifications and the development of cholangiopathies is exemplified in PSC. Indeed, 75% of PSC patients are also affected with inflammatory bowel disease (IBD), more often ulcerative colitis (UC), whose pathogenesis is thought to be linked to gut dysbiosis [16,100,101]. Clinical observations have shown that PSC patients display blood and bile bacteria colonization [102,103] and an increase of 16 S ribosomal RNA (rRNA) in the bile [104]. Intriguingly, colectomy after or during liver transplantation, reduced PSC recurrence (which occurs in up to 37% of PSC-transplanted patients), reflecting the role of gut microbiota in PSC relapse [105,106]. Oral antibiotics have been shown to be effective in PSC patients’ treatment [87,107].

A possible pathogenic role of bacterial translocation has also been proposed for PBC. Lipoteichoic acid, a component of bacterial wall, has been detected in damaged bile ducts of PBC patients [108]. Moreover, long-term exposure of BALB/c mice to bacterial antigens induces autoantibody production which determines histological features resembling PBC [109]. Different risk loci involved in immune regulation and immune response have been identified and genetic susceptibility seems to contribute to chronic biliary diseases establishment [110,111,112]. 

In particular, GWASs have evidenced a correlation between PSC and IBD that share, among others, the genetic risk locus FUT2. The gene FUT2 encodes for galactoside 2-alpha-l-fucosyltransferase 2 enzyme involved in the synthesis of soluble A and B blood antigens. Inactivating variants of FUT2, which define patients as “non-secretors”, influence biliary bacterial diversity with a reduction of *Proteobacteria* and *Actinobacteria*, the enrichment of dominant microbial flora (e.g., *Firmicutes*) and the increase of biliary tree infection rate and incidence of dominant stenosis [113,114]. Knock-out of Fut2 expression in mice has been shown to cause hepatobiliary abnormalities related to the development of porto-systemic shunts [115]. 

The impact of gut microbiota in chronic biliary inflammation has been examined in distinct animal models displaying gut dysbiosis or subjected to bacterial-derived antigens inoculation. In both cases, hepatic changes recapitulating different hallmarks of cholangiopathies, have been reported. Germ-free (GF) mdr2^−/−^ mice model showed a worse biliary injury and higher rate of senescent cholangiocytes, as compared to control mdr2^−/−^ mice conventionally raised [116]. The potential protective role of commensal flora metabolites in the setting of biliary inflammation has been confirmed in vitro. Treatment of senescent cholangiocytes with UDCA, a microbial metabolite absent in GF mice, induced a partial reversal of the senescent phenotype [116]. In line with these findings, decreased levels of secondary bile acids, which are commensal flora metabolites exerting anti-inflammatory functions in vitro, have been found in PSC and IBD patients compared to healthy controls [116]. This is not the case of PBC [116]. Contrasting results have been reported in GF-NOD.c3c4 mice, a mouse model that develops spontaneously intra and extra-hepatic biliary tree inflammation. GF-NOD.c3c4 mice are protected from disease development when compared to conventionally raised (CONV-R) NOD.c3c4. Likewise, biliary disease improvement could be achieved by antibiotic-treatment of CONV-R NOD.c3c4. [117]. The potential role of intestinal luminal bacteria in hepatobiliary inflammation has been explored in genetically susceptible rats, which develop small-intestine bacterial overgrowth (SIBO) after the surgical jejunal self-filling of blind loops. Portal inflammation and bile duct proliferations and loss have been reported in this animal model [118]. The pathological phenotype could be prevented by treatment with Mutanolysin, a muralytic enzyme which breaks the β1-4 linkage of *N*-acetylmuramyl acid and *N*-acetylglucosamine of peptidoglycan [118].

Others in vivo studies rely on bacteria or microbial-derived components animal inoculation to trigger hepatobiliary inflammation. These models resemble various characteristic of diseases of the biliary tree. A clear example derives from an experimental study carried out on BALB/c mice subjected to long-term inoculation of *Streptococcus intermedius*. The prolonged bacterial exposure determines a non-suppurative cholangitis and the production of autoantibodies, resembling histological features of PBC [109]. Moreover, intraperitoneal injection of peptidoglycan in rats causes cholangiographic irregularities of both small and large ductules, focal areas of sacculations and loss of bile mass [119]. Rectal inoculation of *N*-formyl l-methionine l-leucine l-tyrosine (fMLT), an *E. coli*-chemotactic peptide that reaches the liver via portal blood circulation, resulted in small duct cholangitis with leukocytes infiltration, inducing bile duct abnormalities similar to those observed in the earlier stages of PSC [120]. The link between enteric microbes and inflammatory cholangiopathies has been observed in mice lacking the expression of functional CFTR (CFTR^−/−^) and subjected to dextran sodium sulfate (DSS) colitis model [121,122]. DSS treatment increases the intestinal permeability and induces portal endotoxemia [123]. DSS-treated CFTR^−/−^ mice exhibit signs of hepatic injury including the activation of ductular reaction, the establishment of portal inflammation and the infiltration of immune cells, as compared to wild-type (WT) counterpart [124]. In vitro, LPS challenge induces the increase of inflammatory cytokines releasing in CFTR^−/−^ isolated cholangiocytes with respect to WT cultured cells, via upregulation of TLR4 and NF-kB pathway [124]. Taken together these experimental findings support the involvement of microorganisms, PAMPs or bacterial products in the development and progression of cholangiopathies. Nonetheless, it is not yet clear the precise mechanism through which gut-derived products determine biliary tree damage and the degree of injury. Intestinal dysbiosis, loss of intestinal epithelial barrier functions or a deregulated and exacerbated host immune responses are all mechanisms proposed as causal links in the development and progression of chronic biliary inflammatory diseases.

## 5. Microbiota Modification in Cholangiopathies

Several attempts have been made in recent years to define the gut microbiota signature of a group of cholangiopathies. Advances in high-throughput technologies allowed a deeper understanding of gut microbiota composition and modification in the setting of biliary inflammatory diseases. The composition of the microbiota harboring the intestine is the result of different variables such as patient genotype, eating habits, age, health conditions and antibiotic assumption [95]. Typically, the microbiota observed in fecal samples of healthy individuals is mainly enriched of two phyla, *Firmicutes* and *Bacterioides*. *Proteobacteria* accounts for up to 2–3% of gut milieu [125]. Commensal flora possess functional role in nutritive metabolism, by synthetizing nutrients in absorbable substrates for the host, in drugs or xenobiotics metabolism, in intestinal mucosal barrier integrity maintenance, in immune system development and in preventing pathogen bacteria colonization [126].

Multiple sequencing studies have been carried out to precisely characterize the microbiota composition of patients with chronic cholestatic liver diseases, both at fecal and mucosal level. 

A recent study highlighted the existing differences in fecal microbiota composition between PSC-patients and both IBD and healthy patients [127]. At the genus level, PSC patients, with or without concomitant IBD, had increased abundance of *Enterococcus*, *Lactobacillus*, *Streptococcus*, and *Fusobacterium* genera compared to the healthy group. Oral antibiotic treatment has been shown to be effective in *Streptococcus* abundance abolishment [127]. The remaining three genera, independently from oral assumption of UDCA and after removal of confounding factors such as the correlation with IBD, set a “PSC-microbiota signature”, which allowed disease diagnosis with a 71% of accuracy [127]. The overrepresentation of *Enterococcus*, *Lactobacillus*, and *Fusobacterium* genera has been already reported in different diseases such as liver cirrhosis and IBD [128,129,130]. In particular *Enterococcus faecalis*, which along *Enterococcus faecium* is the most represented species revealed in PSC-patients bile with dominant strictures, has been linked to intestinal barrier perturbation due to its ability to produce enzymes belonging to matrix metalloproteases (MMPs) such as gelatinase (GelE) [131]. A positive correlation between *Enterococcus* abundance and increased serum levels of the alkaline phosphatase (ALP), a cholestatic marker associated to a poor prognosis in cholestatic liver diseases, has been reported. However, the results were not confirmed after eliminating confounding factors [127]. 

Additional experimental evidences confirmed a distinct microbiota composition of PSC independently to IBD co-presentation, compared to control group. Sequencing data obtained from stool samples revealed the reduction of alpha diversity and the modification of different bacterial taxa richness in PSC patients with respect to controls. The abundance of *Veillonella*, which belongs to the phylum *Firmicutes*, resulted highly enriched in PSC patients compared to both hepatocellular carcinoma (HCC) and UC patients [132]. Interestingly, the reduced alpha diversity observed in PSC patients as compared to HCC patients is not influenced by oral antibiotics administration in the last 12 months [132]. Thus, the lowering of intra-individual microbiota diversity seems to be a unique characteristic of PSC and not of other liver diseases [129]. *Veillonella*, which has been found to be the most abundant genus enriched in PSC patients, has been previously linked to others inflammatory and fibrotic diseases (pulmonary cystic fibrosis or idiopathic pulmonary fibrosis) and to Crohn’s Disease (CD) relapse after ileo-caecal resection [132]. Accordingly, PSC gut microbiota profile is not influenced by IBD type, suggesting the concept that the dysbiosis is determined by liver disease [132].

With regard to pediatric PSC-patients dysbiosis, an increased abundance of *Streptococcus parasanguinis*, *Veillonella* spp., *Enterococcus faecium* and *Enterococcus* spp. has been evidenced compared to HCCs [133]. The overall findings suggest the association of three major genera, *Enteroccocus*, *Streptococcus*, and *Veillonella* to pediatric PSC-development. The alteration of gut microbiota has been also investigated in fecal samples of PBC patients [134]. The sequencing of 16S rRNA of microbiota performed in stool samples of PBC patients prior to UDCA treatment has shown a reduction in microbiota diversity with respect to the healthy group [134]. Contrasting results have been reported in a separate study, probably due to UDCA treatment of PBC patients selected for the study [135]. As in PSC, a microbial signature consisting of 12 genera has been identified that could be hypothetically used for PBC diagnosis [134]. Microbiota profile analysis highlighted the enrichment of the *Enterobacteriaceae* family, followed by *Pseudomonas*, *Veillonella*, and *Clostridium* genera in PBC patients. Conversely, *Oscillospira* and *Sutterella* resulted as less represented [136]. Six months of UDCA treatment reverted gut microbiota alteration in six of the 12 genera associated to PBC with respect to healthy group [136].

The interaction between gut microbiota and host takes play at intestinal mucosa level suggesting that the suitable way to explore dysbiosis, proposed as the basis of “leaky-gut hypothesis”, is represented by mucosal microbiota profiling. In a recent study, 16S rRNA microarray analysis carried out in ileocecal biopsies collected by 12 PSC patients, evidenced differences in mucosa-adherent microbiota composition with respect to UC (11 individuals) or non-inflammatory patients (nine individuals) [137]. Microbiota profiling showed lower abundance of uncultured *Clostridiales* II, members of *Firmicutes*, in PSC patients compared to both UC and control patients, and a significant reduction of microbiota diversity and abundance in PSC patients with respect to healthy group [137]. A correlation between *Firmicutes* and the health state has been previously reported [138]. The mucosa-adherent microbiota profiling has been performed from ileocecal biopsies collected by 20 PSC patients with (19 individuals) or without IBD (one patient only), 15 IBD-affected individuals and nine healthy controls [139]. Despite the results showed no differences in microbiota composition throughout the ileum or cecum mucosa, an increased abundance of *Blautia* and *Barnesiellaceae* genera and a shift under *Clostridiales* order have been reported in PSC patients [139]. These data are in contrast with a previous study that showed a reduction of uncultured *Clostridiales* in PSC patients [137].

## 6. Conclusions

Intense research over recent years has unveiled many aspects of the pathophysiology of chronic biliary diseases. Cholangiocytes have shifted from passive bystander of cholangiopathies to the central cells orchestrating the complex cellular and molecular interactions occurring in response to damage. Gut-derived bacterial products elicit the activation of strong immune responses in cholangiocytes, which, in genetically susceptible individuals, may contribute to the development or sustain biliary injury. Moreover, disturbances of the intestinal barrier function, with or without intestinal inflammation, may favor the uncontrolled permeation of bacterial products form the gut to the portal circulation, thus perpetuating biliary inflammation. In this context, a specific microbiota composition in patients affected by cholangiopathies is emerging in recent studies and could in part be responsible for cholangiocyte activation. A deeper understanding of the complex relations between biliary inflammation, cholangiocytes response to injury and the role of gut-derived antigens in the pathogenesis of cholangiopathies may prove essential to devise novel effective therapeutic strategies.
